# Magnetoreception in laboratory mice: sensitivity to extremely low-frequency fields exceeds 33 nT at 30 Hz

**DOI:** 10.1098/rsif.2012.1046

**Published:** 2013-04-06

**Authors:** Frank S. Prato, Dawn Desjardins-Holmes, Lynn D. Keenliside, Janice M. DeMoor, John A. Robertson, Alex W. Thomas

**Affiliations:** 1 Bioelectromagnetics Group, Imaging Program, Lawson Health Research Institute, London, Ontario, CanadaN6A 4V2; 2 Department of Imaging, St. Joseph's Health Care, London, Ontario, CanadaN6A 4V2; 3 Department of Medical Biophysics, Schulich School of Medicine and Dentistry, Western University, London, Ontario, CanadaN6A 5C1

**Keywords:** magnetic field sensitivity, ambient magnetic field shielding, nociception, analgesia, extremely low-frequency magnetic fields, geomagnetic static field

## Abstract

Magnetoreception in the animal kingdom has focused primarily on behavioural responses to the static geomagnetic field and the slow changes in its magnitude and direction as animals navigate/migrate. There has been relatively little attention given to the possibility that weak extremely low-frequency magnetic fields (wELFMF) may affect animal behaviour. Previously, we showed that changes in nociception under an ambient magnetic field-shielded environment may be a good alternative biological endpoint to orientation measurements for investigations into magnetoreception. Here we show that nociception in mice is altered by a 30 Hz field with a peak amplitude more than 1000 times weaker than the static component of the geomagnetic field. When mice are exposed to an ambient magnetic field-shielded environment 1 h a day for five consecutive days, a strong analgesic (i.e. antinociception) response is induced by day 5. Introduction of a static field with an average magnitude of 44 µT (spatial variability of ±3 µT) marginally affects this response, whereas introduction of a 30 Hz time-varying field as weak as 33 nT has a strong effect, reducing the analgesic effect by 60 per cent. Such sensitivity is surprisingly high. Any purported detection mechanisms being considered will need to explain effects at such wELFMF.

## 1. Introduction

The literature on the effects of geomagnetic fields on animal orientation and homing has focused primarily on the response to the static field rather than the potential for a response to comparatively weak extremely low-frequency magnetic fields (wELFMF). Although there is a wELFMF ‘jitter’ on the static field associated with solar activity and thunderstorms (e.g. Schumann resonances [1]), it is normally of the order of a nanotesla. Hence, it is unlikely that such random weak fields affect animal orientation and homing, except perhaps by being disruptive during intense solar activity and thunderstorms, when values can exceed 100 nT. Of more relevance is the observation that birds and other animals can orient using the geomagnetic field to a precision of approximately 1° [2–4]. This would correspond to the ability to detect wELFMF of the order of tens of nanotesla.

There have been a few attempts to establish a threshold for behavioural effects of wELFMF. Kirschvink *et al.* [5], using a food reward experiment, established that the minimal fields detectable by honeybees were 4.3 µT at 10 Hz and 430 µT at 60 Hz. Nishimura *et al.* [6] reported that effects on tail lifting behaviour could be observed in a species of lizards at 6 and 8 Hz for a peak field of 2.6 µT. In a study by Burda *et al.* [7], disruptive effects on alignment of ruminants by a 50 Hz field from high voltage electric power lines continued out to a distance between 20 and 100 m, where the field strengths are between 0.1 and 1 µT. Burger *et al.* [8] reported that a 1 µT 0.5 Hz magnetic field affected c-Fos expression, a measure of neuronal activity, in the navigation circuit in Ansell's mole-rats. Further, Ossenkopp *et al.* [9] observed that opioid-related behaviours in laboratory mice (CF-1) were affected on 17 December 1982 when there was a significant increase in the degree of geomagnetic disturbance (from Ap index of 11 to 62 corresponding to approx. 120 nT). There have also been reports [10,11] that an underground ELF antenna generating 0.1–0.5 µT between 72 and 80 Hz can disrupt bird orientation, which supports an account of effects of geomagnetic disturbances on orientation in birds [12].

Although most of the work on animal navigation, homing and orientation has focused on non-mammalian animals, there have been more recent studies indicating that mammals, and specifically rodents, can also detect the geomagnetic field. In addition to the c-Fos work of Burger *et al.* [8], others have reported that modification of the direction of the static component of the geomagnetic field affects location of behaviours such as nest-making and sleeping [13–16]. In addition, there is growing evidence that laboratory rodents (Siberian hamster [17] and C57Bl/6J mice [18]) can be taught behaviours that can be affected when exposed to altered static magnetic field components of the geomagnetic field. However, except for the work by Burger *et al*., all of these studies ignored the non-static magnetic field components, i.e. they failed to report and account for the wELFMF that could have been present owing to geomagnetic disturbances or anthropogenic sources of 50 or 60 Hz magnetic fields. In this regard, it is of interest that Oliveriusova *et al.* [16] point out that orientation of mole-rats in a rotated geomagnetic field exhibit much higher scatter of bearings as compared with orientation in the ‘natural’ magnetic field. Could this be caused by a ‘natural’ wELFMF that was not rotated? Hence, we have proposed that thresholds to the sensing of static and wELFMF should be carried out under magnetic field shielding conditions where such fields can be carefully introduced without confounds of exposure to ambient wELFMF.

Here we show, using a laboratory mouse model in which magnetic sensitivity may be driven by the same mechanism as that for bird orientation and homing [19,20], that sensitivity to tens of nanotesla does occur. This is consistent with the concept that animals capable of using the geomagnetic field for orientation and homing perceive the spatial variation in the static field as an induced wELFMF as the animal traverses that spatial variation. Alternatively, the wELFMF variation in a projected constant field sensed by a ‘fixed’ receptor could also be perceived as the animal changes its orientation by a degree or two.

We have established that laboratory-bred mice, when exposed to an ambient magnetic field-shielded environment for 1 h per day for five consecutive days, develop a strong antinociceptive response by the fifth day [21]. This response is probably opioid-mediated and is sensitive to the intensity and wavelength of light [20], similar to some aspects of bird navigation [22]. Using this experimental protocol, we estimated in a preliminary pilot experiment in 20 animals that nociceptive behaviour effects may be detected for 30 Hz fields possibly as low as 30 nT [19]. Here, we show that (i) the effect at 30 Hz, 30 nT is robust, because it has been confirmed in 120 animals, (ii) the induction of analgesia is definitely not related to the elimination of ambient electric fields, and (iii) the induction of analgesia may also be partially attenuated by the introduction of a static field of geomagnetic amplitude (44 µT ± spatial variability of 7%). Earlier work [19] suggested an induced current mechanism based on the observation that the size of the effect of the introduced ELF magnetic fields was dependent on the product of the ELF frequency and the ELF magnitude. However, how such weak fields could induce sufficient current remains a mystery [23]. In addition, other proposed magnetoreception mechanisms, such as those based on radical pairs [24] or arrays of iron particles [25–27], are also theoretically challenged by sensitivities down to tens of nT.

## 2. Material and methods

### 2.1. Animals

Adult male Swiss CD-1 mice (Charles River, St. Constant, Quebec, Canada) two to four months old and weighing 25–35 g were used. Mice were housed individually in polycarbonate cages under a 12 L : 12 D cycle at 22 ± 2°C. Food and water were freely available.

### 2.2. Assessment of nociception

Nociception (pain sensitivity) was measured as the latency of a foot lifting/lick to an aversive thermal stimulus (hot plate test; model HP AccuScan Instruments, Inc., Columbus, OH) at 50 ± 0.5°C. The maximum individual latency observed was 45 s; hence, all mice were removed from the heated surface before the cut-off time of 60 s set by the Animal Use Subcommittee.

### 2.3. Magnetic exposure conditions

Extremely low-frequency magnetic fields inside the mu-metal boxes (0.20–0.35 µT static, less than 0.001 µT 60 Hz) were attenuated more than 50 times [28] in comparison with ambient fields (from static to 125 Hz). The magnitude of the magnetic field as a function of frequency in the animal housing room and for the testing room has been previously reported [19]. The static field was approximately 45 µT in magnitude, and the ELF spectrum shows peaks at 60, 120, 180 and 240 Hz, which combined have an amplitude of approximately 0.15 µT. The mu-metal boxes are identical to the one described by Koziak *et al.* [29]. The boxes (33 × 38 × 20 cm) were made of 1.6 mm (1/16 in) thick mu-metal (Magnetic Shield Corp., Bensenville, IL or Amuneal Manufacturing Corp., Philadelphia, PA), with magnetically shielded holes (diameter of 2.5 cm) at each of the four corners (1 cm off the sides) of both the base and top surfaces. A mu-metal cylinder (2.5 cm high) surrounded each hole, shielding the ambient magnetic field and light. The mu-metal boxes were laminated inside with black opaque polyethylene, as it is impervious to virtually all solvents. Individual mice were placed in a 26 × 16 × 12 cm clean transparent polycarbonate cage and covered by a clear polycarbonate (Lexan) top with ventilation holes (diameter of 8 mm). This cage was inserted into the mu-metal box. Individual mice are free to move within the polycarbonate cage in the mu-metal box. A sham box, identical to the dimensions of the mu-metal boxes, was constructed of opaque fibreglass material and was also lined with opaque polyethylene. The sham box had no effect on the ambient magnetic field. In addition, a box made of stainless steel (Amuneal Manufacturing Corp) was added that did not attenuate magnetic fields below 250 Hz by more than 3 per cent but was expected to significantly attenuate the ambient ELF electric field. This stainless steel box was identical in appearance and similar in weight to the mu-metal boxes.

A set of four coils was placed inside all boxes except the fibreglass sham box. The coils were made of 150 turns of AWG #28 magnet wire (0.32 mm diameter with a thin layer of enamel insulation), and were put on plastic rectangular formers with mean dimensions of 30 × 17.8 cm. The DC resistance was approximately 31 *Ω* on each former. The four coils were spaced 9.7 cm apart in a Merritt-like configuration for a total length of 29 cm on centre. The coils were connected to each channel of a four-channel controlled current amplifier. To minimize the introduction of an electric field, the coils were electrically shielded with conductive silver paint and copper foil. Connection leads were routed through the magnetically shielded holes at the top rear corner of the mu-metal enclosure and connected to the controlled current amplifier. Each coil carries approximately 3.787 × 10^−4^ A peak to deliver a 500 nT peak field. The coils are nominally 31 *Ω* each (single wound). Calculated power is (3.787 × 10^−4^)^2^ × 31 = 4.446 × 10^−6^ W per coil or 1.778 × 10^−5^ W for the four coils. For sinusoidal operation, this value is multiplied by 0.707 giving an apparent power of 1.257 × 10^−5^ W_RMS_. It is unlikely that heating is a confound in our 30 Hz experiments because the maximum ELF field used in these experiments was 65 nT. In contrast, the power needed to produce the 44 µT static field (approx. 0.1 W) is equivalent to about a third of the metabolic rate of a 30 g mouse (approx. 0.34 W). Note that within these coils, the magnetic field varied by ±7% over the volume occupied by the mouse cage.

The signal generator was an in-house arbitrary function generator configured to produce either 64-step waveforms for frequencies above 51 Hz or 512-step waveforms for lower frequencies. Using a Tektronix Inc. (Beaverton, OR) TDS3032 oscilloscope (FFT Log scale Hanning window), the second harmonic was measured at −56 dB, the third at −49 dB and the fourth at −56 dB of the fundamental frequencies for a 100 Hz 64-step sinusoidal pattern. The 6.4 kHz fundamental of the 64 steps for a 100 Hz sinusoidal signal was also measured. The second harmonic is 41 dB below the 100 Hz fundamental, and harmonics are seen up to the 20th at about −72 dB (just visible above noise). Note that, for the introduction of a static field, a ripple at ELF frequencies would be attenuated by more than 70 dB, as the 60 Hz ripple on the ±15 V amplifier is approximately 1 per cent and the output op amps have a typical power supply rejection ratio of 69 dB.

Amplitude calibration was done by selecting sets of current limiting resistors and adjusting the amplifier gain control to achieve a desired field strength, as measured by a Bartington 3-axis fluxgate magnetometer (MAG-03MS 1000, Bartington Instruments, Oxford, UK) with a full-scale waveform pattern. Subsequent lower fields were obtained by changing the software: dividing the waveform pattern amplitudes by the required factor and uploading as needed. Field strength was measured as peak values. The accuracy of our MAG-03MS 1000 magnetometer was checked by comparison with our MAG-03MC 500 unit for which we have a calibrated source (MAG-03MC-CU). No drift in the accuracy of the unit could be detected.

Frequency calibration was achieved by selection of appropriate waveform patterns and numerical calculation of the point latency. Output frequency was then confirmed with both a Fluke 179 Multimeter (Fluke Electronics Canada, Mississauga, ON, Canada) and a Tektronix TDS3032 oscilloscope.

Acoustic properties inside the test enclosures were checked with a Brüel & Kjær (DK-2850 Nærum, Denmark) one-inch condenser microphone (model 4131) and preamplifier (model 2801). Output was measured with the oscilloscope using FFT Log scale Hanning window and showed no detected signal outside of normal ambient background.

### 2.4. Lighting conditions

Because we have shown that the induced analgesia can be attenuated/abolished by simultaneous light exposure, care was taken to eliminate light intensity and wavelength as confounders. Light levels in all boxes were below the sensitivity of the spectrophotometer (LightSpex; McMahan Research Laboratories, Chapel Hill, NC) and certainly below 2.0 × 10^16^ photons s^−1^ m^−2^.

### 2.5. Experimental procedures

Three experiments were undertaken: the first to confirm the effects of re-introduction of a 65 nT, 30 Hz field; the second to establish that a 33 nT, 30 Hz field was above the detection threshold and the third to investigate the effect of the introduction of a static field of the same average magnitude as the ambient geomagnetic static field, but with a spatial variability of ±7% over the volume in which the mice could traverse. Note that all stated values of ELF magnetic field amplitude are peak values of the sinusoidal fields.

#### 2.5.1. Experiment 1

For five consecutive days, each mouse (*N* = 240) was exposed to one of four conditions (for 1 h) and tested pre- and post exposure (hot plate latency in seconds). The time of day of the exposure was randomized between 09.00 and 15.00. The exposure conditions used were sham (fibreglass enclosure with no ELFMF shielding), stainless steel enclosure without activation of inserted coils, positive control (mu-metal enclosure providing static and ELFMF shielding without activation of inserted coils) and five mu-metal enclosures with active coils at 30 Hz sinusoidal with a magnetic field amplitude at 65 nT. Note that there were 30 animals per exposure condition; however, animals were exposed singly.

#### 2.5.2. Experiment 2

This was identical to experiment 1 except that four of the five experimental exposure boxes were set at 33 nT, with the fifth at 65 nT. As in experiment 1, there were 30 animals per exposure condition for a total of 240 animals.

#### 2.5.3. Experiment 3

This only used four boxes. The fibreglass sham, the stainless steel control and the mu-metal positive control were identical to those in experiments 1 and 2. The fourth box was mu-metal with the coil energized to produce an average horizontal static field of 44 µT with a variation of ±7% over the volume occupied by the mouse cage set within each box. Note there were 40 animals per exposure condition for a total of 160.

In order to keep the experiments double-blinded regarding exposure conditions, the signal generators did not display the amplitude or pattern selection and only L.D.K. (a technical manager separate from the actual experiments) knew the conditions. While D.D.H. carried out the experiments, A.W.T. completed the analysis prior to L.D.K. disclosing the exposure conditions.

### 2.6. Data analysis

For each of the three experiments, the data were analysed using an omnibus mixed design analysis of variance (ANOVA; IBM SPSS Statistics v. 19.0, USA) investigating the relationships between day (5, repeated), pre- versus post exposure (2, repeated) and condition (8, independent, for experiments 1 and 2; 4, independent, for experiment 3). Parametric post hoc analysis using Tukey's honestly significant difference (HSD) examined the differences between sets of data. Further parametric post hoc analysis (single-tailed paired *t*-test) compared post-exposure latency times on days 4 and 5 for each of the five 30 Hz exposures in experiments 1 and 2 with the positive control (0 nT) and with the sham control (fibreglass box) for the respective experiments. In experiment 3, days 4 and 5 of the 44 µT exposed animals were similarly compared with the positive control (0 nT) and the sham control (fibreglass box).

A third analysis was performed to determine the fractional reduction in analgesia that could be attributed to the sinusoidal or static magnetic fields introduced into the mu-metal box. This was done by calculating the sum of the differences between post- and pre-latencies for days 3, 4 and 5 for each of the five groups in experiments 1 and 2 and for the single exposure group in experiment 3. This sum was subtracted from the sum for the respective positive control for each experiment. This difference was then divided by the summed latencies for the respective positive control. The result corresponds to the fractional decrease in induced analgesia for days 3, 4 and 5 caused by the introduced magnetic field. This provided a total of 11 outcomes, six at 65 nT, four at 33 nT and one for the static field.

## 3. Results

**Figure 1.**
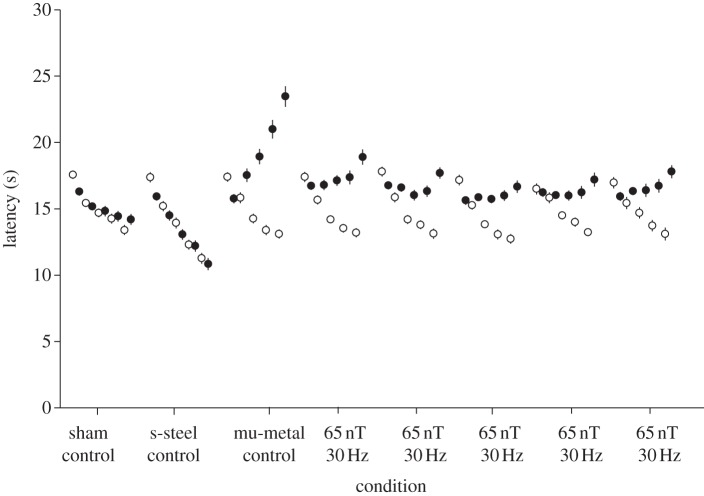
Pre- (open circles) and post-exposure (filled circles) latencies for 30 Hz, 65 nT as well as fibreglass sham, stainless steel control and 0 nT (mu-metal positive control). The five entries for each of the eight conditions refer to the five experimental days. Error bars correspond to ±standard error of the mean (s.e.m.). Where s.e.m. bars are not evident, they fall within the symbols. The ANOVA analysis showed a significant three-way interaction between day (5, repeated) by pre–post (2, repeated) by condition (8, independent) (*F*_2,28_ = 5.30, *p* < 0.001, **η**^2^ = 0.14).

**Figure 2.**
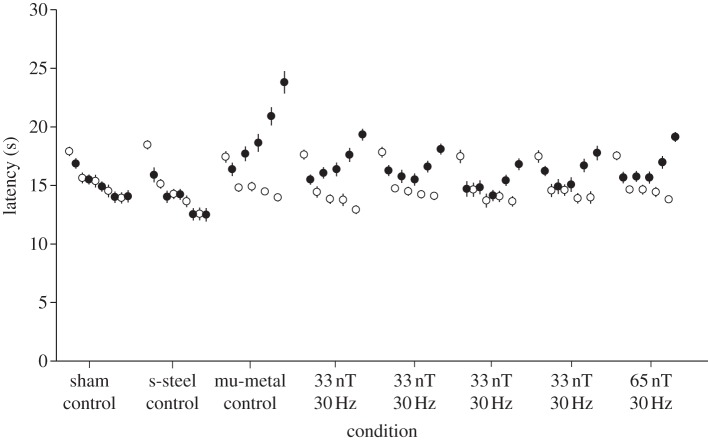
Pre- (open circles) and post-exposure (filled circles) latencies for 30 Hz, 65 nT and four 30 Hz, 33 nT exposures as well as a fibreglass sham, a stainless steel control and a 0 nT mu-metal positive control. The five entries for each of the eight conditions refer to the five experimental days. Error bars correspond to±standard error of the mean (s.e.m.). Where s.e.m. bars are not evident, they fall within the symbols. The ANOVA analysis showed a significant three-way interaction between day (5, repeated) by pre–post (2, repeated) by condition (8, independent) (*F*_2,28_ = 4.00, *p* < 0.001, **η**^2^ = 0.11).

**Figure 3.**
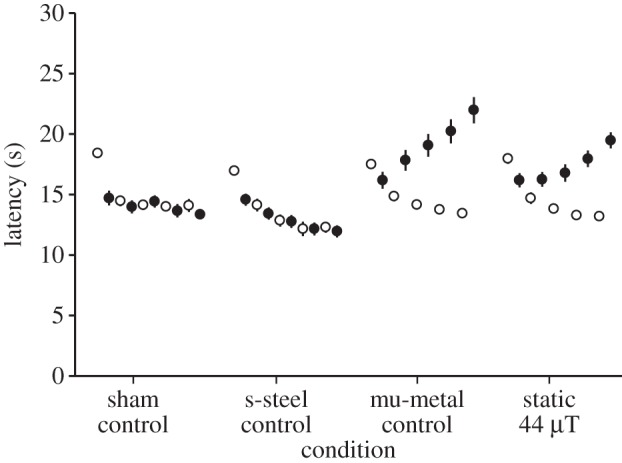
Pre- (open circles) and post-exposure (filled circles) latencies for a 44 µT static field exposure as well as a fibreglass sham, a stainless steel control and a 0 nT mu-metal positive control. The five entries for each of the four conditions refer to the five experimental days. Error bars correspond to ±standard error of the mean (s.e.m.). Where SEM bars are not evident, they fall within the symbols. The ANOVA analysis showed a significant three-way interaction between day (5, repeated) by pre–post (2, repeated) by condition (4, independent) (*F*_2,38_ = 5.98, *p* < 0.001, **η**^2^ = 0.13).

Figures 1–3 show the results of experiments 1, 2 and 3. For all three experiments, the ANOVA analysis (details in the respective figure captions) showed a highly significant three-way interaction. Post hoc Tukey's HSD analyses of homogeneity for sets of data across conditions are shown in tables 1–3 for experiments 1–3, respectively. Table 4 provides the significance values calculated for the post hoc comparisons for days 4 and 5 and the fractional decreases summed over days 3, 4 and 5 in the induced analgesia.

**Table 1.** Post hoc Tukey's HSD analysis of homogeneity for sets of data across conditions for experiment 1. (Means for groups in homogeneous subsets are displayed. The error term is m.s.e. = 2.200).

exposure condition	*n*	subset		
		1	2	3
s-steel control	30	13.6600		
sham control	30		15.0300	
65 nT, 30 Hz	30		15.1967	
65 nT, 30 Hz	30		15.5867	
65 nT, 30 Hz	30		15.7133	
65 nT, 30 Hz	30		15.8233	
65 nT, 30 Hz	30		16.0933	16.0933
mu-metal control	30			17.0700
significance		1.000	0.106	0.180

**Table 2.** Post hoc Tukey's HSD analysis of homogeneity for sets of data across conditions for experiment 2. (Means for groups in homogeneous subsets are displayed. The error term is m.s.e. = 3.490).

exposure condition	*n*	subset		
		1	2	3
s-steel control	30	14.3267		
33 nT, 30 Hz	30	14.9400	14.9400	
sham control	30	15.2700	15.2700	
33 nT, 30 Hz	30	15.5167	15.5167	
33 nT, 30 Hz	30	15.7467	15.7467	
33 nT, 30 Hz	30	15.7633	15.7633	
65 nT, 30 Hz	30		15.8233	15.8233
mu-metal control	30			17.2967
significance		0.063	0.599	0.051

**Table 3.** Post hoc Tukey's HSD analysis of homogeneity for sets of data across conditions for experiment 3. (Means for groups in homogeneous subsets are displayed. The error term is m.s.e. = 6.413).

exposure condition	*n*	subset		
		1	2	3
s-steel control	40	13.3250		
sham control	40	14.5225	14.5225	
static 44 µT	40		15.9600	15.9600
mu-metal control	40			16.8975
significance		0.153	0.058	0.351

**Table 4.** Single-tailed post hoc significance (*p*) values comparing post-exposure latency times on days 4 and 5 in magnetic field-exposed mice to the respective positive (0 nT) and sham (fibreglass) controls, and the induced fractional attenuation in induced analgesia (exp, experiment; freq, frequency; ampl, amplitude).

exp. no.	freq. (hz)	peak ampl. (nT)	nT-Hz	statistical significance (*p*-value)				fractional attenuation in induced analgesia
				at 4 days	at 4 days	at 5 days	at 5 days	
				to 0 nT	to sham	to 0 nT	to Sham	
exp. 1	30	65	1950	<0.0001	<0.0001	<0.0001	<0.0001	0.449
	30	65	1950	<0.0001	0.0010	<0.0001	<0.0001	0.605
	30	65	1950	<0.0001	0.0040	<0.0001	<0.0001	0.613
	30	65	1950	<0.0001	0.0020	<0.0001	<0.0001	0.655
	30	65	1950	<0.0001	0.0005	<0.0001	<0.0001	0.585
exp. 2	30	65	1950	<0.0001	<0.0001	<0.0001	<0.0001	0.554
	30	33	990	0.0004	<0.0001	<0.0001	<0.0001	0.361
	30	33	990	<0.0001	0.0002	<0.0001	<0.0001	0.631
	30	33	990	<0.0001	0.0200	<0.0001	0.0002	0.752
	30	33	990	<0.0001	0.0003	<0.0001	<0.0001	0.646
exp. 3	0	44 000	0	0.0320	<0.0001	0.0260	<0.0001	0.305

For experiment 1, the introduction of the 65 nT, 30 Hz exposure reduced the induction of analgesia observed on the third to fifth days of testing versus that in the positive control by 58 ± 3%. For experiment 2, 33 nT, 30 Hz reduced the induction of analgesia seen in the positive control by 60 ± 8%. In experiment 3, the static field reduced the induction of analgesia on the third to fifth days of testing versus that in the positive control by 30.5 per cent. As expected in all three experiments, the sham exposure did not induce analgesia. The stainless steel box, anticipated to shield only the ELF electric fields, also did not induce analgesia in any of the experiments (figures 1–3).

The latency times post-exposure on days 4 and 5 were significantly different (*p* < 0.05) as compared with the mu-metal control and the sham control for all three experiments (see table 4).

## 4. Discussion

The results of experiment 1 (figure 1) indicate an attenuation in induced analgesia of 58 per cent for an exposure of 65 nT at 30 Hz (peak amplitude frequency product of 1950 nT-Hz) (table 4). This is consistent with fractional attenuation values of 0.44 for 1500 nT-Hz (at 30 Hz) and 0.60 for 3000 nT-Hz (at 30 Hz) previously reported by our group [19]. Results from experiment 2 (figure 2) indicate an attenuation in induced analgesia of 60 per cent for an exposure of 990 nT-Hz (table 4), which seems high based on previous work. However, this recent work is based on an experiment that included a total of 120 mice, whereas the previous work only had results for 20 mice. In figure 2, relatively large effects are seen at 33 nT, 30 Hz, which strongly suggests the threshold of detection is below 1000 nT-Hz! Additional experiments further reducing peak field are needed to titrate the threshold at 30 Hz and to determine any dependence of that threshold with frequency.

Experiment 3 (figure 3) suggests for the first time that this experimental protocol may detect effects of exposure to a static field with a variation of ±3 µT. Previous experiments had suggested that the induction of analgesia was not associated with the shielding of the static geomagnetic field. But, in that earlier work [28], the Plexiglass box was placed within three orthogonally nested coils that zeroed the static ambient field but not the ELF field, and the attenuation of stress-induced analgesia was investigated for a single 2 h exposure. Note that these square Helmholtz-like coils were approximately 2 m in diameter and hence had relatively little spatial variation over the dimensions corresponding to the space available for mouse movement. Here we have re-introduced only the magnitude of the local static ambient field. Note that an effect of 30.5 per cent attenuation in induced analgesia is of interest. In previous work [19], we showed that the maximum attenuation in induced analgesia through the introduction of ELF magnetic fields was 70.7 per cent. The combination of the effects of the static field (30.5%) and the ELF field (70.7%) equals approximately 100 per cent attenuation and, therefore, we speculate that this may explain the entire phenomenon.

There is a major potential confound to the interpretation of experiment 3. It is entirely possible that the mice were exposed to an apparent ELF magnetic field owing to movement of the mice within the spatial variation in this static field, i.e. variations as large as±3 µT. If this turns out to be correct, then the effect produced by this ‘apparent’ ELF is at a higher amplitude but much lower frequency than the 33 nT, 30 Hz exposures. Experiments to resolve this question should be undertaken where (i) the gradient is reduced, and/or (ii) the gradient is increased while maintaining the same average static field of 44 µT.

An effect of Earth-strength static magnetic fields on laboratory mice could involve at least two mechanisms currently being debated in the animal orientation/homing literature: a radical pair [24] and/or arrays of iron particles made of magnetite [27]. However, as presently modelled, it is difficult to understand how these mechanisms could explain effects of wELFMF of 33 nT peak at 30 Hz. Our earlier work suggested effects consistent with an induced current. However, the formulation of such a mechanism based on induced currents faces formidable challenges associated with background tissue noise [23]. Further, the results reported here cast doubt on our earlier hypothesis that effects near the threshold vary as the amplitude frequency product.

With respect to iron particles, Kirschvink *et al.* [5] proposed that the detection mechanism for magnetoreception in bees is due to iron particles that hold a permanent magnetic dipole. Experiments done by Walker & Bitterman [30] suggest that the median threshold sensitivity to a static field is 260 nT but they report one bee with a sensitivity down to 26 nT. Kirschvink *et al.* [5] explored bee sensitivity to a 10 and 60 Hz field and determined thresholds of 4.3 and 430 µT, respectively. This is clearly far in excess of 33 nT. However, their experimental set-up requires the bees to travel through a strong magnetic field gradient under ambient magnetic field conditions. Polk [26] and Adair [25] estimated a threshold of 5 µT at 60 Hz for a single magnetic magnetite particle, which seems low compared with the results of the bee work. In addition, arrays of iron particles could increase sensitivity and one-dimensional arrays are clearly used and effective in magnetotactic bacteria [31]. If we estimate an improvement in signal to noise proportional to the square root of the number of particles in the array, then approximately 10 000 would be needed to allow a threshold of 50 nT, rather than 5 µT. In addition, if the particles were too small or too large to hold a permanent field, then an array of such iron magnetite particles could also explain effects that have been reported that do not discriminate between the Earth's magnetic poles. Note that the very important recent work of Wu & Dickman [27] demonstrates convincingly that there is a neurosubstrate for magnetic field detection including polarity, direction and magnitude of a static field. But their conjecture that the detector is a permanent magnetic particle is simply based on speculation driven by alternate neuronal firing when the static field is rotated 180°. In their paper they report a static magnetic field threshold of 20 µT. Again, experiments are conducted under ambient wELFMF conditions. It would be important to see the effect on neuronal firing in (i) a shielded environment and (ii) under wELFMF in a shielded environment.

With respect to the free radical biophysical detection mechanism, the main experimental indications are light sensitivity/dependency and an inclination compass (meaning the inability to detect the Earth's magnetic field polarity). However, an alternative explanation is an array of non-magnetic particles and the need for light to trigger an essential post-detection event. The attractiveness to some is that requirements of coherence and entanglement of a free radical pair would usher in a quantum biology effect [24,32,33]. But, in a recent theoretical analysis, Hogben *et al*. [34] suggest that coherence and entanglement are, in fact, not needed. The authors argue that lifting this requirement ‘offers new and more flexible opportunities for the design of biologically inspired magnetic compass sensors’. However, the radical pair mechanism is certainly challenged to explain thresholds of 33 nT at 30 Hz without prolonged coherence and entanglement. Sensitivity to an ELF frequency may in fact be a property of a ‘downstream’ biological event that determines whether or not the initial detection results in a behavioural/physiological response. This would eliminate the need of the radical pair to be sensitive to magnetic fields over a number of milliseconds, and hence the dependence on a prolonged coherence and entanglement time would no longer be required.

In the non-orientation bioelectromagnetics literature, there has been a data-supported theory that ion–protein complexes can detect wELFMF [35]. The associated initial transduction theories require specific resonances between the ELF frequency, the ELF amplitude and the relative orientation of the ELF field vector, and that of a ‘superimposed’ static field [36,37]. It is of interest that these theories were heavily criticized [38,39] in that the oscillating metal ion in the potential well could not achieve coherence long enough to detect an ELFMF. Binhi [40] has recast this model to address these issues and has fit theoretical curves to the data from a number of the cyclotron resonances investigators (see especially figs 2.11, 4.32, 4.45). Also, Lednev's work should not be discounted given his prediction, supported by experimental data, that effects are expected under zeroed static field conditions [36,37]. Note that similar criticisms are currently an issue with respect to the radical pair mechanism [41]. However, the evidence associated with orientation, migration and homing has become so convincing in recent years that some are suggesting that quantum coherence of the spin states must be preserved even under ‘warm and wet’ conditions [32]. Perhaps, the progress being made in solid-state physics with respect to spin quantum memory [33] may provide clues as to how spin coherence lifetime can be extended. Such an extension would imply isolation from the ‘thermal bath’, but would also need to address issues of frequency preference and the very low detection threshold established herein. A better establishment of the sensitivity threshold of wELFMF and dependence on frequency would help validate these models and allow a calculation of the needed radical pair coherence time.
